# Preserved corneal lamellar transplantation for infectious and noninfectious scleral defects: Three case reports and literature review

**DOI:** 10.1097/MD.0000000000026607

**Published:** 2021-07-23

**Authors:** Young-ho Jung, Chang Ho Yoon, Mee Kum Kim

**Affiliations:** a Department of Ophthalmology, Seoul National University College of Medicine, 103 Daehak-ro, Jongno-gu, Seoul, Republic of Korea; b Department of Ophthalmology, Seoul National University Hospital, 101 Daehak-ro, Jongno-gu, Seoul, Republic of Korea; c Laboratory of Ocular Regenerative Medicine and Immunology, Biomedical Research Institute, Seoul National University Hospital, 101 Daehak-ro, Jongno-gu, Seoul, Republic of Korea; d Transplantation Research Institute, Seoul National University Medical Research Center, 103 Daehak-ro, Jongno-gu, Seoul, Korea.

**Keywords:** acellular corneal patch graft, case report, preserved corneal lamellar transplantation, scleral defect, scleritis

## Abstract

**Rationale:**

: Reinforcement of thinned or necrotizing sclera has been conducted using various materials, including allogeneic sclera, allogenic cornea, amniotic membrane, fascia lata, pericardium, periosteum, and perichondrium. Among them, good outcomes have traditionally been obtained using preserved scleral grafts. However, scleral patch grafts have complications such as graft retraction, thinning, dehiscence, and necrosis. Furthermore, to promote epithelial healing, scleral patch grafting must be accompanied by procedures such as amniotic membrane transplantation (AMT) or grafting using conjunctival flaps or autografts. Recently, acellular preserved human corneas have been used in various ophthalmic surgeries, with emerging evidence supporting its use for treating scleral defects as an option that does not require AMT or conjunctival autografting. We investigated whether corneal patch grafting would show wound healing and tectonic success rate outcomes comparable to those of existing techniques.

**Patient presentation:**

: Three patients presented with intractable ocular pain. Slit-lamp examination showed marked scleral thinning at the nasal side.

**Diagnosis:**

: Scleral thinning progressed with conservative treatment; microbial staining and culturing were performed. Infectious or non-infectious scleritis was diagnosed according to slit-lamp examination and microbial culture results.

**Interventions:**

: A preserved corneal lamellar patch was grafted at the scleral thinning area.

**Outcomes:**

All patients achieved tectonic success with reduction of inflammation following corneal patch grafting. Two patients achieved complete re-epithelialization within 7 days, while 25 days were required for the third patient. No patients experienced graft thinning, rejection, or infection.

**Lessons:**

: Our report suggests the feasibility of using acellular preserved human cornea patch grafts to reinforce inflammatory scleral defects and obtain successful outcomes in terms of wound healing. This technique shows a comparable tectonic success rate and superior effect on scleral defect healing without the need for adjunctive AMT or conjunctival autografting.

## 1 Introduction

Infectious or noninfectious scleral necrosis with calcium deposition could occur after pterygium removal, trauma, idiopathic systemic vasculitis, or retinal detachment surgery.^[1]^ Severe scleral thinning with exposed calcium or uveal tissue in the defect area can induce secondary infection or necrotizing scleritis and leave the eyeball vulnerable to minor trauma.^[2]^ To prevent these adverse events, scleral reinforcement is performed to cover the thinned sclera using different materials, including allogeneic sclera, amniotic membrane, fascia lata, pericardium, periosteum, and perichondrium.^[3–5]^ Among them, good outcomes have traditionally been obtained using preserved scleral grafts. However, these are associated with complications such as graft retraction, thinning, dehiscence, and necrosis.^[6]^ Furthermore, scleral graft transplantation must be accompanied by amniotic membrane transplantation (AMT) to promote epithelization over the scleral or conjunctival graft,^[7,8]^ which increases surgical time.^[9]^ Acellular preserved human corneal tissue can be transplanted to the scleral defect area without the need for AMT or conjunctival grafting, which has been shown to reduce inflammation and accelerate wound healing in rabbits.^[10]^ Compared with the sclera, the cornea has a basement membrane and collagen fibrils that are arranged compactly, which is expected to be beneficial for wound healing and graft survival.^[11]^

We report on 3 cases of infectious or noninfectious scleral defects with successful transplantation of acellular preserved corneal lamellar grafts followed by rapid wound healing and inflammation reduction. This technique seems to have an effect on scleral graft healing comparable or superior to that of adjunctive AMT or conjunctival autograft.

## 2 Case presentation

### 2.1 Case 1

**Figure 1:**
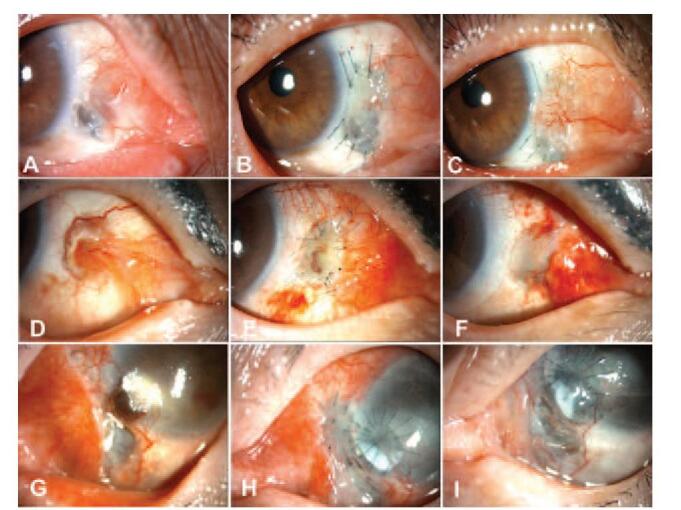
Representative photos of 3 cases with corneal patch grafts A, B, C: Case 1. (A): Preoperative scleral thinning with calcification. (B): One month postoperatively. (C): One year postoperatively. D, E, F: Case 2. (D): Preoperative scleral thinning with conjunctival injection and calcification. (E): One week postoperatively. (F): One month postoperatively. G, H, I: Case 3. (G): Preoperative scleral thinning with calcification and impending perforation. (H): One month postoperatively. (I): Six months postoperatively.

A 74-year-old female underwent right-eye pterygium surgery approximately 13 years ago. She presented to our ophthalmology department with a six-month history of right ocular pain (Table 1). Her right-eye best corrected visual acuity (BCVA) was 20/20. Marked scleral thinning with a 4.5 × 4.5 cm calcium plaque was observed medially on slit-lamp examination (Fig. 1A). No organism was isolated on microbial staining and culture. The patient was diagnosed with noninfectious scleritis following pterygium removal surgery. A preserved corneal lamellar patch (Halo; Eversight Inc., Palo Alto, CA) was grafted after calcium plaque excision. At 1 week postoperatively, ocular surface epithelialization was complete and inflammation was diminished. At 1 month postoperatively, vascularization had partially progressed with regression of inflammation (Fig. 1B). One year postoperatively, the graft had been vascularized and a stable surface had been maintained (Fig. 1C). Her BCVA remained at 20/20.

**Table 1:** Overview of clinical characteristics in present cases.

		Preoperative				Postoperative		
No	Sex/Age	BCVA	Previous Op.	Finding	Cultured organism	BCVA	HT	FUT
1	74 / W	20/20	Pterygium op.	Scleromalacia with calcium plaque	Negative	20/20	7D	12 m
2	66 / W	20/20	None	Scleral melting with calcium plaque	Bacillus	20/20	7D	1 m
3	82 / M	4/20	None	Scleral melting, impending perforation	Bacillus Paecilomyces	LP	25D	8 m

BCVA = best corrected visual acuity, FUT = follow up time, HT = healing time, LP = light perception, No = number.

### 2.2 Case 2

A 66-year-old female with ocular pain was referred to our department (Table 1). Her right-eye BCVA was 20/20. On slit-lamp examination, scleral thinning with infection and a 5 mm × 3.2 mm exposed calcium plaque was observed on the nasal aspect of the right eye (Fig. 1D). Although the patient received anti-inflammatory medication before referral, scleral thinning had progressed. *Bacillus* species was isolated on microbial staining and culture. The patient was diagnosed with infectious scleritis with exposed calcium plaque. The calcium plaque was excised, and a preserved corneal lamellar graft (Halo; Eversight Inc., Palo Alto, CA) was transplanted. One week postoperatively, with topical application of 0.5% moxifloxacin (Vigamox, Alcon, TX), the graft surface was fully epithelized and her BCVA was 20/20 (Fig. 1E). One month postoperatively, the graft surface was stable and there was no infection or inflammation (Fig. 1F). Subconjunctival haemorrhages were noted after suture removal (Fig. 1F).

### 2.3 Case 3

An 82-year-old male visited our hospital with a three-month history of severe pain and decreased visual acuity in the left eye (Table 1). At the initial visit, his BCVA was 4/20. Slit-lamp examination revealed diffuse scleral infection, nasal scleromalacia with a large calcified plaque, an inferotemporal scleral nodule, and nasal corneal stromal oedema with endothelial infiltration. Severe anterior chamber inflammation was also observed. The nodule was filled with pus originating from the nasal side. Culture revealed *Paecilomyces* and *Bacillus* species. The patient was diagnosed with fungal scleritis with bacterial infection. Hourly topical administration of 0.5% moxifloxacin (Vigamox, Alcon Laboratories, Inc., Fort Worth, TX), 2.5% fortified vancomycin, 5% ceftazidime, and 1% voriconazole, as well as oral administration of 200 mg voriconazole twice daily and 400 mg moxifloxacin once daily was performed.

Five weeks later, although the infection was partially controlled, scleral necrosis had worsened compared to the initial visit. The defect size was 5 × 8 mm with iris prolapse at the inferonasal limbus that extended to the scleral defect (Fig. 1G). After calcium plaque (4 × 9 mm) excision, the patient underwent tectonic keratoplasty using a full thickness corneoscleral graft and patch grafting using preserved corneal tissue (Halo; Eversight Inc., Palo Alto, CA). Twenty five days postoperatively, inflammation was reduced and complete graft epithelialization was observed (Fig. 1H). Six months postoperatively, the graft was vascularized and stable (Fig. 1I). Although his BCVA was only light perception, infection had subsided and the eyeball was saved.

## 3 Discussion

Surgical management of infectious or noninfectious scleritis with severe thinning remains challenging, and scleral wall reinforcement has been attempted using various materials.^[3–5,12]^ We used an acellular preserved human cornea patch graft to manage scleral thinning with inflammation and obtained successful wound healing and inflammation reduction outcomes. In a retrospective report of 13 cases of necrotizing scleritis, scleral thinning, or dehiscence by Sangwan et al, the tectonic success rate was 76.9% and 3 complications were noted (endophthalmitis, graft necrosis, and graft dehiscence). In addition, re-epithelization occurred after an average of 3 to 4 weeks.^[6]^ Oh et al reported that the scleral graft remained intact and no recurrence was observed in 8 patients with noninfectious scleromalacia. Re-epithelization was observed within 7 days in 7 of those patients; 6 weeks were required for re-epithelization in the remaining patient.^[9]^ Kim et al reported a high success rate of grafting using autologous perichondrium with AMT in 17 out of 18 eyes (94.4%) at 6 months postoperatively. However, their surgical technique necessitates harvesting the perichondrium from the tragus prior to AMT, which increases surgical time.^[5]^

A literature review reveals that our tectonic success rate and re-epithelialization time were comparable to those previously reported in studies of scleral grafting (Table 2); moreover, there were no adverse events. The shorter surgical time may be a benefit of our technique over scleral grafting combined with adjunctive surgeries such as AMT, conjunctival autograft/flap use, or perichondrial harvesting. Given that corneal type 4 collagen facilitates corneal epithelial adhesion, faster wound healing may occur with corneal grafting.^[13]^ In Case 3, epithelialization took a relatively long time (25 days). Considering that it was a case of fungal infection with a poor prognosis, this could be regarded as a relatively good outcome. The patient's decreased visual acuity was due to corneal opacity and iris synechiae obscuring the pupil.

**Table 2:** Overview of previously published studies on scleral or corneal graft to reinforece scleral defect^∗^.

Graft	Author	No.	Diagnosis	Adjunctive procedure	Mean HT (weeks)	Mean FUT (months)	Success, %	Complication^†^, %
Sclera^‡^	Sangwan VS^[6]^	13	Necrotizing scleritis Scleromalacia	Conjunctival flap or AMT	3.5	24.3	76.9	23.1
	Oh JH^[20]^	8	Scleromalacia	AMT	1	24	100	0
Fresh Donor Cornea	Naseripour M^[12]^	4	Necrotizing scleritis	None	NA	28.5	100	0
	Ti SE^[14]^	20	Infectious scleritis	Conjunctival flap	NA	14	95	5^‡^
	Kymionis GD^[15]^	1	Necrotizing scleritis	None	2	12	100	0
	Lin HC^[16]^	1	Infectious scleritis	Conjunctival flap	NA	24	100	0
	Ramos-Esteban^[17]^	1	Infectious scleritis	Pericardium	NA	16	100	0
Acellular preserved cornea	Huang CY^[18]^	1	Necrotizing scleritis	None	1	12	100	0
	Ram R^[19]^	1	Necrotizing scleritis	None	NA	8	100	0
	Present cases	3	Noninfectious and infectious scleritis	None	2	7	100	0

∗ Results of literature review of articles published after the year 2000.

† Complication: graft dehiscence, graft necrosis, recurrence of infection.

‡ Literatures with more than 3 cases were included. AMT = amniotic membrane transplantation, FUT = follow up time, HT = healing time, NA = not available, No = number of cases.

Several studies of acellular or cellular corneal graft use for scleral reinforcement using various surgical techniques have been published (Table 2); some report acceptable effectiveness of corneal patch grafting in patients with scleral thinning after pterygium surgery, penetrating keratoplasty, or radiotherapy.^[12,14–17]^ However, they used corneal grafts from fresh corneas or donated whole corneas after Descemet stripping endothelial keratoplasty. Two reports of successful tectonic outcome using acellular corneal graft in patients with scleritis following strabismus surgery have been published.^[18,19]^ The rate of epithelialization was mentioned in only one study.^[18]^ Taken together, this study supports the evidence that preserved corneal lamellar grafting may be feasible for scleral defects.

No study directly compares the efficacy of corneal and scleral grafts for treating scleral defects. However, we can infer and compare the effects based on studies in patients who underwent glaucoma valve surgery with corneal grafting. These studies showed comparable tectonic results between corneal and scleral grafts, and they found that surgeons used corneal grafts 3.8 times more frequently than scleral grafts.^[20,21]^ In addition, evidence suggests that acellular preserved corneal grafting is effective for reducing inflammation and promoting wound healing in rabbits.^[10]^

This study was limited by the small number of patients and short follow-up duration. Nevertheless, it provides evidence that acellular preserved corneal grafting promotes wound healing, has comparable tectonic success rates to those of traditionally used techniques, and may have the advantage of a shorter surgical time in the treatment of scleral defects. Therefore, surgeons should consider acellular preserved cornea grafting as an option for the treatment of infectious or noninfectious scleral defects.

## Author contributions

**Conceptualization:** Mee Kum Kim.

**Data curation:** Mee Kum Kim.

**Formal analysis:** Young Ho Jung.

**Methodology:** Mee Kum Kim.

**Supervision:** Mee Kum Kim.

**Validation:** Mee Kum Kim.

**Visualization:** Young Ho Jung, Chang Ho Yoon.

**Writing – original draft:** Young Ho Jung, Mee Kum Kim.

**Writing – review & editing:** Young Ho Jung, Chang Ho Yoon, Mee Kum Kim.

## References

[1] Riono WP Hidayat AA Rao NA. Scleritis: a clinicopathologic study of 55 cases. Ophthalmology 1999;106:1328–33. 10406616 10.1016/S0161-6420(99)00719-8

[2] Galor A Thorne JE. Scleritis and peripheral ulcerative keratitis. Rheum Dis Clin North Am 2007;33:835–54. 18037120 10.1016/j.rdc.2007.08.002 PMC2212596

[3] Mauriello JA Pokorny K. Use of split-thickness dermal grafts to repair corneal and scleral defects--a study of 10 patients. Br J Ophthalmol 1993;77:327–31. 8318477 10.1136/bjo.77.6.327 PMC504524

[4] Koenig SB Kaufman HE. The treatment of necrotizing scleritis with an autogenous periosteal graft. Ophthalmic Surg 1983;14:1029–32. 6672701

[5] Kim JT Kim KW Mun SK Chun YS Kim JC. Transplantation of autologous perichondrium with amniotic membrane for progressive scleral necrosis. Ocul Surf 2019;17:571–7. 31112741 10.1016/j.jtos.2019.05.004

[6] Sangwan VS Jain V Gupta P. Structural and functional outcome of scleral patch graft. Eye (Lond) 2007;21:930–5. 16601740 10.1038/sj.eye.6702344

[7] Kim BH. Surgical treatment of necrotic scleral calcification using combined conjunctival autografting and an amniotic membrane inlay filling technique. Eye (Lond) 2011;25:1484–90. 21869832 10.1038/eye.2011.209 PMC3213665

[8] Kim JC Tseng SC. Transplantation of preserved human amniotic membrane for surface reconstruction in severely damaged rabbit corneas. Cornea 1995;14:473–84. 8536460

[9] Oh JH Kim JC. Repair of scleromalacia using preserved scleral graft with amniotic membrane transplantation. Cornea 2003;22:288–93. 12792468 10.1097/00003226-200305000-00002

[10] Kim KW Ryu JS Kim JY Kim MK. Preserved corneal lamellar grafting reduces inflammation and promotes wound healing in a scleral defect rabbit model. Transl Vis Sci Technol 2020;9:38 doi:10.1167/tvst.9.7.38. 10.1167/tvst.9.7.38 PMC7414681 32832243

[11] Torricelli AA Singh V Santhiago MR Wilson SE. The corneal epithelial basement membrane: structure, function, and disease. Invest Ophthalmol Vis Sci 2013;54:6390–400. 24078382 10.1167/iovs.13-12547 PMC3787659

[12] Naseripour M Aghaei H Sedaghat A Kheirkhah A Jaberi R Azma Z. Corneal patch graft: a new approach for scleral necrosis secondary to plaque radiotherapy. Cornea 2016;35:565–8. 26863496 10.1097/ICO.0000000000000716

[13] Cameron JD Skubitz AP Furcht LT. Type IV collagen and corneal epithelial adhesion and migration. Effects of type IV collagen fragments and synthetic peptides on rabbit corneal epithelial cell adhesion and migration in vitro. Invest Ophthalmol Vis Sci 1991;32:2766–73. 1894474

[14] Ti SE Tan DT. Tectonic corneal lamellar grafting for severe scleral melting after pterygium surgery. Ophthalmology 2003;110:1126–36. 12799236 10.1016/S0161-6420(03)00260-4

[15] Kymionis GD Plaka AD Diakonis VF. Cross-linking as an adjuvant treatment for tectonic corneal lamellar graft preparation. Open Ophthalmol J 2013;7:79–81. 24278090 10.2174/1874364101307010079 PMC3837367

[16] Lin HC Ma DH Chen YF Yeh LK Hsiao CH. Late-onset intrascleral dissemination of Stenotrophomonas maltophilia scleritis after pterygium excision. Cornea 2011;30:712–5. 21173698 10.1097/ICO.0b013e31820007ed

[17] Ramos-Esteban JC Servat JJ Silva RS Ambrosio R Tauber S Bia F. Necrotizing nocardial scleritis after combined penetrating keratoplasty and phacoemulsification with intraocular lens implantation: a case report and review of the literature. Arq Bras Oftalmol 2007;70:355–9. 17589714 10.1590/s0004-27492007000200031

[18] Huang CY Lin HC Yang ML. Necrotizing scleritis after strabismus surgery in thyroid eye disease. J AAPOS 2013;17:535–6. 24160978 10.1016/j.jaapos.2013.04.010

[19] Ram R. Tectonic corneal lamellar grafting for surgically-induced necrotizing scleritis after strabismus surgery: case report & literature review. Am J Ophthalmol Case Rep 2018;11:28–31. 30062145 10.1016/j.ajoc.2018.04.025 PMC6063979

[20] Passo RM Hoskins ZB Tran KD. Electron beam irradiated corneal versus gamma-irradiated scleral patch graft erosion rates in glaucoma drainage device surgery. Ophthalmol Ther 2019;8:421–6. 31172462 10.1007/s40123-019-0190-x PMC6692416

[21] Spierer O Waisbourd M Golan Y Newman H Rachmiel R. Partial thickness corneal tissue as a patch graft material for prevention of glaucoma drainage device exposure. BMC Ophthalmol 2016;16:20 doi:10.1186/s12886-016-0196-2. 26920383 10.1186/s12886-016-0196-2 PMC4769544

